# Quantitative Assessment of Bone Density at the Borders of Radiolucent Mandibular Lesions Using Cone-Beam Computed Tomography: Correlations With Lesion Aggressiveness

**DOI:** 10.7759/cureus.86111

**Published:** 2025-06-16

**Authors:** Rukshana Begum, Rupali Malik, Khushbu Gupta, Prabhjeet Kaur, Monika Bhat, Ishita Garg, Manish Sharma

**Affiliations:** 1 Department of Oral Medicine and Radiology, Kalinga Institute of Dental Sciences, Bhubaneswar, IND; 2 Department of Public Health Dentistry, Teerthanker Mahaveer Dental College and Research Centre, Moradabad, IND; 3 Department of Oral Medicine and Radiology, Index Institute of Dental Sciences, Indore, IND; 4 Department of Oral Medicine and Radiology, Kothiwal Dental College and Research Centre, Moradabad, IND; 5 Department of Oral Medicine and Radiology, Adesh Institute of Dental Sciences and Research, Bathinda, IND; 6 Department of Oral Pathology, Jawahar Medical Foundation's Annasaheb Chudaman Patil Dental College, Dhule, IND

**Keywords:** bone density, cone-beam computed tomography, lesions, mandible, radiolucent

## Abstract

Introduction: Radiolucent mandibular lesions present diagnostic challenges owing to their diverse nature, ranging from benign cysts to aggressive neoplasms, with varying impacts on the surrounding bone. This study aimed to evaluate bone density variations at the borders of these lesions using cone-beam computed tomography (CBCT) and to correlate these findings with histopathological indicators of lesion aggressiveness to identify quantitative imaging markers for improved diagnosis and treatment planning.

Materials and methods: A retrospective cross-sectional study was conducted, involving 125 patients with histopathologically confirmed unilocular or multilocular radiolucent mandibular lesions. High-resolution CBCT scans (voxel size ≤0.2 mm) were acquired using a standardized protocol, and bone density was measured in Hounsfield Units (HU) at the anterior, posterior, inferior, superior, buccal, and lingual borders. Regions of interest (1 mm³) were systematically placed with adjacent normal bone as a reference, and measurements were averaged from three repetitions by two trained observers to ensure reliability (intraclass correlation coefficient of 0.87). Descriptive statistics, the Mann-Whitney U test, and linear regression (p < 0.05) were used to analyze bone density differences and associations with lesion characteristics.

Results: Significant bone density variations were noted, with inferior regions showing the highest density (1557.92 HU) and buccal regions the lowest (605.2 HU). Aggressive lesions and the absence of root resorption were strongly associated with reduced bone density across all regions (p < 0.001), except in the buccal region (p = 0.256). The absence of cortical perforation increased buccal density (p < 0.001) but reduced density elsewhere (p ≤ 0.027). Unilocular lesions correlated with higher density in the posterior, superior, and lingual regions (p ≤ 0.024), whereas smooth-bordered lesions were associated with lower density in most regions (p ≤ 0.002) but higher buccal border density (p = 0.035). Female sex was associated with a reduced density at the inferior border (p = 0.001).

Conclusion: These findings suggest that CBCT-based bone density measurements can differentiate lesion aggressiveness, with regional variations reflecting biomechanical and pathological interactions, potentially guiding targeted diagnostic and therapeutic strategies.

## Introduction

Radiolucent mandibular lesions encompass a diverse group of pathological entities, ranging from benign cysts to aggressive neoplasms, each presenting unique diagnostic and therapeutic challenges [1]. The analysis of radiolucent lesions in the mandible can pose significant difficulties, either because of the nonspecific nature of the clinical manifestations or the possibility that the lesions are identified incidentally [2]. These lesions, often identified on routine dental imaging, vary in their clinical behavior, with aggressiveness influencing treatment planning and prognosis [3]. An accurate assessment of lesion characteristics, particularly at their borders, is critical for determining their nature and guiding clinical management.

Standard radiographic examinations of the mandible, commonly referred to as orthopantomograms, may reveal lesions characterized by radiolucent, radiodense, or mixed patterns [3]. In numerous instances, such as radicular cysts, the diagnostic process is uncomplicated, necessitating no further imaging for accurate diagnosis and subsequent treatment [2]. Given that traditional radiographs represent two-dimensional projections of three-dimensional (3D) anatomical entities, their efficacy is constrained by the evaluation of lesion dimensions, borders, and the extent of involvement of critical anatomical structures or surrounding soft tissues [4]. Cone-beam computed tomography (CBCT) has emerged as a pivotal imaging modality in maxillofacial radiology due to its high-resolution, 3D visualization capabilities, which offer superior detail compared to conventional radiography [4,5]. High-resolution CBCT with a thin slice thickness of 1 mm and optimized bone window settings is predominantly employed in the preoperative phase to meticulously evaluate the dimensions, margins, destructive characteristics, and expansion patterns of lesions, as well as to analyze the spatial relationship between the lesion and the mandibular canal [4,6].

Bone density at lesion borders, quantifiable through CBCT gray values (Hounsfield units, HU), may reflect the lesion’s interaction with the surrounding bone, potentially indicating its aggressive potential [7]. Previous studies have suggested that variations in bone density at lesion margins correlate with histopathological features of aggressiveness, such as cortical erosion or rapid expansion [8]. However, standardized protocols for quantifying these changes and their association with lesion behavior remain underexplored. According to a study by Cassetta et al. [9], a highly significant positive correlation exists between the CBCT voxel value (VV) and computed tomography (CT) HU values.

This study aimed to evaluate bone density variations at the borders of radiolucent mandibular lesions using CBCT and to correlate these findings with lesion aggressiveness, as confirmed by histopathology. By employing a retrospective cross-sectional design, we sought to establish a quantitative imaging technique that could aid in differentiating benign from aggressive lesions to ultimately improve diagnostic accuracy and treatment outcomes.

## Materials and methods

Study design and setting

This retrospective cross-sectional analytical study was conducted from January 2025 to March 2025 on CBCT records of the patients from the dental radiology department of the Kothiwal Dental College and Research Center, Moradabad. CBCT records of five years were considered for this study from January 2020 to January 2025. The ethical clearance was obtained from the Institutional Ethical Review Board (IERB) of the host institution (KDCRC/IERB/12/2024/S130). All procedures complied with the ethical principles outlined in the Declaration of Helsinki, ensuring patient confidentiality through anonymization of all data prior to analysis.

Sample size estimation

The sample size was determined using G*Power version 3.1 (Heinrich-Heine-Universität Düsseldorf, Düsseldorf, Germany) based on a pilot study (n = 15), indicating an effect size of 0.5, bone density differences between lesion borders and adjacent normal bone, with a power of 80% and a significance level of 5% (α = 0.05), yielding an initial sample size of 113 patients, adjusted to 125 patients to account for a 10% attrition rate due to potential nondiagnostic CBCT scans such as motion artifacts or incomplete field of view (FOV), focusing on patients with well-defined unilocular or multilocular radiolucent mandibular lesions confirmed by both CBCT imaging and histopathological examination.

Patients’ eligibility

Patients were included if they had radiographically confirmed unilocular or multilocular radiolucent mandibular lesions with histopathological verification (such as odontogenic cysts, ameloblastomas, or other benign or malignant lesions), presence of CBCT scans with high resolution (voxel size ≤ 0.2 mm) and a complete FOV encompassing the lesion and surrounding bone, while excluding poor-quality scans with motion artifacts, scatter, or incomplete FOV; mixed-density lesions with both radiolucent and radiopaque components; patients with prior surgical intervention or trauma in the mandibular region; systemic bone disorders such as osteoporosis, osteomalacia, or fibrous dysplasia; patients under 18 or over 80 years to minimize age-related bone density variations; and pregnant patients due to radiation exposure risks, ensuring a homogeneous sample for reliable imaging and histopathological data.

Methodology

High-resolution CBCT scans were acquired using a Carestream CS 9300 (Carestream Health, Inc., Rochester, NY) with a standardized protocol optimized for mandibular imaging, using a limited FOV (5 × 5 cm or 8 × 8 cm) based on lesion size to minimize radiation exposure while capturing the entire lesion and adjacent bone, a voxel size of ≤0.2 mm for precise bone density assessment, a tube voltage of 90 kVp to balance image quality and radiation dose, a variable tube current (4-10 mA) adjusted based on patient size to optimize contrast and reduce artifacts, and a scan time of approximately 12-20 seconds, with multiplanar reconstructions (axial, coronal, and sagittal views) generated using Carestream 3D software (Carestream Health, Inc.) to facilitate 3D analysis of lesion borders and surrounding bone, performed by a trained oral and maxillofacial radiologist, with daily calibration of the CBCT machine using a CIRS Model 062M phantom (CIRS Inc., Norfolk, VA) to verify gray values accuracy and HU conversion. The lesions were classified into aggressive and nonaggressive types based on the lesion border following the criteria given by Lodwick et al [10].

**Figure 1:**
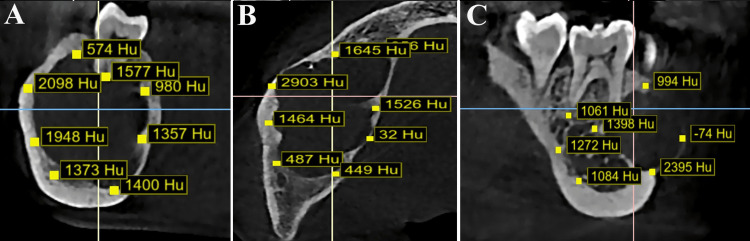
Cone-beam computed tomography images of mandible. Bone density (Hounsfield unit as Hu) measurements in nonaggressive mandibular radiolucent lesion: (A) oblique plane, (B) axial plane, and (C) sagittal plane Image credit: all images are original and related to case studies

**Figure 2:**
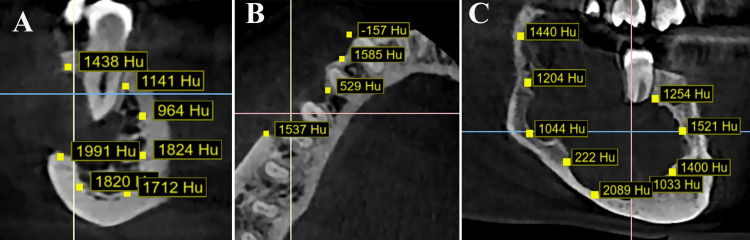
Cone-beam computed tomography images of mandible. Bone density (Hounsfield unit as Hu) measurements in aggressive mandibular radiolucent lesion: (A) oblique plane, (B) axial plane, and (C) sagittal plane Image credit: all images are original and related to case studies

Digital Imaging and Communications in Medicine-formatted CBCT volumes were imported into BlueSkyPlan software (Blue Sky Bio, LLC, Grayslake, IL) for semiautomated bone density assessment, where rectangular regions of interest (ROIs) of 1 mm³ were systematically placed at the lower, upper, anterior, and posterior borders of each lesion, with adjacent normal bone (1 mm away from the lesion margin) serving as a reference to account for individual bone density variations (Figures 1, 2).

Mean gray values (in HU) were recorded for each ROI. Measurements were repeated three times per border by two independent trained observers with over five years of experience (Rukshana Begum, Monika Bhat) to ensure reproducibility, averaging the results to minimize variability. A third observer (Khushbu Gupta) resolved discrepancies if interobserver differences exceeded 5%. All the observers were from different institutions to avoid observer bias and were provided with coded CBCT data. Prior to data collection, a standardized training session using 20 CBCT scans unrelated to the study was conducted to align ROI placement techniques, achieving an initial intraclass correlation coefficient (ICC) of 0.85 (95% confidence interval, CI: 0.80-0.89), indicating high interobserver reliability, with monthly recalibration of the Carestream CS 9300 and BlueSkyPlan software ensuring consistent accuracy throughout the study.

Reliability was ensured through rigorous calibration of Carestream CS 9300 using the CIRS Model 062M phantom before each imaging session to standardize HU measurements, with BlueSkyPlan software calibrated to manufacturer specifications for consistent gray value output. After a two-week interval, 10% of the CBCT scans were reevaluated by trained observers to assess inter- and intraobserver reliabilities. Each observer independently remeasured the bone density at the lesion borders using BlueSkyPlan software, with measurements repeated three times per ROI. The ICC for interobserver reliability was 0.87 (95% CI: 0.82-0.91), and the intraobserver reliability yielded ICC values of 0.90 (95% CI: 0.86-0.93) for the first observer and 0.89 (95% CI: 0.85-0.92) for the second, indicating excellent reliability.

Statistical analysis

Data were analyzed using SPSS version 26.0 (IBM Corp., Armonk, NY), with descriptive statistics (mean ± standard deviation) summarizing bone density values across lesion borders, and frequencies and percentages provided as categorical data. The normality of the data was checked using the Shapiro-Wilk test. As the data were found to be nonnormally distributed, the Mann-Whitney U test was used to compare bone density among lesion types (e.g., aggressive and nonaggressive). Linear regression was used to identify predictors of aggressive behavior, such as cortical erosion or root resorption, with a p value of <0.05 considered statistically significant. The ICC was calculated to assess inter- and intraobserver reliabilities, targeting an ICC of ≥0.80 for acceptable agreement. The statistician (Ishita Garg) was provided with coded CBCT data.

## Results

Descriptive analysis of 125 cases of radiolucent lesions in the mandible (Table 1) revealed a slight male predominance in women. Odontogenic keratocysts (OKCs) were the most frequent lesions, followed by ameloblastoma, central giant cell granuloma, and dentigerous cysts. Ameloblastic fibroma, adenomatoid odontogenic tumor, and odontogenic myxoma are less common. Aggressiveness and root resorption were observed in 60 (48%) patients, whereas cortical perforation occurred in 30 (24%). Most lesions were unilocular with scalloped borders, indicating varied radiographic presentations and clinical behaviors (Table 1).

**Table 1:** Descriptive analysis of categorical variables in a sample of 125 patients Data are presented in the form of n and %

Variables	Category	Frequency (n)	Percentage (%)
Sex	Female	55	44
	Male	70	56
Type of lesions	Ameloblastic fibroma	10	8
	Ameloblastoma	30	24
	Adenomatoid odontogenic tumor	10	8
	Central giant cell granuloma	20	16
	Dentigerous cyst	15	12
	Odontogenic myxoma	5	4
	Odontogenic keratocyst	35	28
Aggressiveness	No	65	52
	Yes	60	48
Adjacent root resorption	No	65	52
	Yes	60	48
Cortical perforation	No	95	76
	Yes	30	24
Locularity	Multilocular	35	28
	Unilocular	90	72
Borders	Scalloped	80	64
	Smooth	45	36

Descriptive analysis of continuous variables showed a mean age of 29.4 years. Mean bone density values included average bone density at 1077.03 HU, anterior bone density at 1152 HU, posterior bone density at 1248 HU, inferior bone density at 1557.92 HU, superior bone density at 737.52 HU, buccal bone density at 605.2 HU, and lingual bone density at 1161.56 HU. These findings suggested significant variability in bone density across regions, with inferior bone density being the highest and buccal the lowest, potentially influencing lesion behavior and treatment planning (Table 2).

**Table 2:** Descriptive analysis of continuous variables in a sample of 125 patients Data are presented in the form of mean and SD at 95% CI SD: standard deviation; CI: confidence interval; HU: Hounsfield units

Variables	Mean	SD	95% CI mean
Age (years)	29.40	8.34	30.87-27.92
Average bone density (HU)	1,077.03	564.94	1,177.25-976.81
Bone density at the anterior border (HU)	1,152.00	767.96	1,287.95-1,016.05
Bone density at the posterior border (HU)	1,248.00	849.69	1,398.42-1,097.58
Bone density at the inferior border (HU)	1,557.92	875.10	1,712.84-1,403.00
Bone density at the superior border (HU)	737.52	468.05	820.38-654.66
Bone density at the buccal margin (HU)	605.20	546.00	702.06-508.34
Bone density at the lingual margin (HU)	1,161.56	514.12	1,252.58-1,070.54

The Mann-Whitney U test comparing bone density between aggressive and nonaggressive radiolucent lesions revealed significant differences in most variables, with p values of 0.001 for average bone density, anterior bone density, posterior bone density, inferior bone density, superior bone density, and lingual bone density. However, the buccal bone density showed no significant difference (p = 0.256). These findings indicated that aggressive lesions were associated with significantly higher bone density across most regions, suggesting greater bone remodeling or destruction. The lack of significance in buccal bone density might reflect regional variations in lesion impact, potentially guiding targeted diagnosis and treatment approaches (Table 3).

**Table 3:** Comparison of bone density in HU between the aggressive and nonaggressive radiolucent lesions using the Mann-Whitney U test Data are presented in the form of mean and SD ^*^p value <0.05: significant HU: Hounsfield units; SD: standard deviation

Variables (HU)	Nonaggressive, mean ± SD	Aggressive, mean ± SD	Z value	p value
Average bone density	545.87 ± 62.78	1,652.46 ± 137.63	-9.64	0.001^*^
Bone density at the anterior border	454.85 ± 155.35	1,907.25 ± 312.73	33.26	0.001^*^
Bone density at the posterior border	457.69 ± 178.26	2,104.17 ± 222.19	45.86	0.001^*^
Bone density at the inferior border	724.46 ± 63.89	2,460.83 ± 103.40	113.89	0.001^*^
Bone density at the superior border	298.08 ± 54.09	1,213.58 ± 118.40	56.31	0.001^*^
Bone density at the buccal margin	658.69 ± 81.23	547.25 ± 782.83	49.56	0.256
Bone density at the lingual margin	681.46 ± 61.18	1,681.67 ± 149.77	0.01	0.001^*^

Linear regression analysis revealed that various clinical and radiographic factors significantly influenced bone density across different regions. Female sex was associated with reduced inferior bone density (p = 0.001), potentially owing to hormonal or anatomical factors. Aggressive lesions and absence of root resorption were strongly linked to decreased bone density across all regions (p < 0.001), indicating widespread bone loss driven by osteoclastic activity and inflammation. The absence of cortical perforation significantly reduced bone density in the anterior (p = 0.027), posterior (p = 0.012), and lingual (p = 0.006) regions, but increased buccal bone density (p < 0.001), possibly due to reactive bone formation, with a notable impact on overall average bone density (p < 0.001). Unilocular lesions were associated with higher bone density in posterior (p = 0.024), superior (p = 0.005), and lingual (p < 0.001) regions, suggesting less invasive behavior than multilocular lesions. Smooth-bordered lesions, often nonaggressive, were associated with lower bone density in the anterior (p = 0.002), posterior (p < 0.001), inferior (p < 0.001), and lingual (p < 0.001) regions, as well as overall bone density (p < 0.001), but higher buccal density (p = 0.035), possibly due to regional compensatory remodeling. These findings highlighted the complex interplay between lesion characteristics and variations in bone density across different anatomical sites (Table 4).

**Table 4:** Linear regression analysis of bone density with various variables ^*^p value <0.05: significant HU: Hounsfield units

Variables	Bone density at anterior border (HU)		Bone density at posterior border (HU)		Bone density at inferior border (HU)		Bone density at superior border (HU)		Bone density at buccal margin (HU)		Bone density at lingual margin (HU)		Average bone density (HU)	
	t value	p value	t value	p value	t value	p value	t value	p value	t value	p value	t value	p value	t value	p value
Sex (female)	1.75	0.083	0.51	0.614	3.86	0.001^*^	-0.98	0.331	-0.70	0.485	-0.60	0.548	1.46	0.147
Aggressiveness	-21.71	0.001^*^	-32.33	0.001^*^	-86.69	0.001^*^	-40.19	0.001^*^	-35.62	0.001^*^	-35.65	0.001^*^	-67.67	0.001^*^
Root resorption	-21.71	0.001^*^	-32.33	0.001^*^	-86.69	0.001^*^	-40.19	0.001^*^	-35.62	0.001^*^	-35.65	0.001^*^	-67.67	0.001^*^
Cortical perforation (no)	-2.24	0.027^*^	-2.56	0.012^*^	-0.61	0.543	-0.30	0.763	77.2	0.001^*^	-2.78	0.006^*^	11.33	0.001^*^
Locularity (unilocular)	-0.32	0.749	2.29	0.024^*^	1.47	0.144	-2.84	0.005^*^	1.05	0.297	4.01	0.001^*^	1.71	0.091
Border (smooth)	-3.15	0.002^*^	-5.30	0.001^*^	-3.50	0.001^*^	-1.16	0.248	2.13	0.035^*^	-5.09	0.001^*^	-5.95	0.001^*^

## Discussion

This study investigated bone density variations at the borders of radiolucent mandibular lesions using CBCT and correlated these findings with histopathological indicators of lesion aggressiveness. The results provided valuable insights into the interplay between the clinical, radiographic, and histopathological characteristics of mandibular lesions and their impact on surrounding bone density, offering potential imaging biomarkers to enhance diagnostic accuracy and guide treatment planning.

The findings indicated significant regional variations in bone density, with inferior bone density (1557.92 HU) being the highest and buccal bone density (605.2 HU) the lowest among the measured sites. The differences in bone density between the buccal and inferior mandibular regions are likely influenced by a combination of anatomical structures, lesion characteristics, and muscle attachments. The buccal region’s lower baseline density is consistent with its thinner cortical plate, but the masseter’s strong attachment may promote localized bone formation, particularly in nonaggressive lesions or in the absence of cortical perforation [11]. This is evidenced by the study’s finding of increased buccal bone density in specific contexts (p < 0.001), which aligns with Wolff’s law and the role of muscle-induced mechanical stress in bone remodeling [12].

In contrast, the high density of the inferior region reflects its thick cortical structure and load-bearing role, with muscle attachments (such as digastric and mylohyoid) playing a secondary role in maintaining density [11,13]. However, pathological processes, such as aggressive lesions or hormonal influences in females, may override these effects, leading to significant bone loss in the inferior region (p < 0.001). The interplay between muscle attachments and lesion behavior suggests that biomechanical forces from muscles, such as the masseter, can modulate bone density responses, particularly in the buccal region, where compensatory remodeling appears more pronounced [14].

A key finding was the significant association between lesion aggressiveness and reduced bone density across most regions (p < 0.001), except for the buccal bone density (p = 0.256). Aggressive lesions, such as ameloblastomas or OKCs, are associated with widespread bone loss, likely due to osteoclastic activation and inflammatory processes, as supported by prior studies [15,16]. The lack of significance in buccal bone density may be attributed to compensatory bone remodeling or reactive sclerosis, which can occur in response to mechanical stress or lesion expansion, particularly in less aggressive lesions. This regional discrepancy highlights the complexity of lesion-bone interactions and suggests that the buccal bone may serve as a unique indicator of lesion behavior, warranting further investigation.

The absence of root resorption was also strongly associated with reduced bone density across all the regions (p < 0.001). This finding aligns with the understanding that root resorption, often associated with aggressive lesions, triggers significant osteoclastic activity, leading to bone loss [3]. The consistent impact across all regions emphasizes the destructive potential of lesions exhibiting root resorption, necessitating careful monitoring and aggressive treatment strategies to preserve the mandibular integrity.

Interestingly, the absence of cortical perforation was associated with reduced bone density in the anterior (p = 0.027), posterior (p = 0.012), and lingual (p = 0.006) regions, but increased buccal bone density (p < 0.001). This paradoxical increase in buccal density may reflect reactive bone formation, where the buccal cortex undergoes compensatory thickening in response to lesion pressure or mechanical stress, as suggested in previous studies [17]. The significant impact on the overall average bone density (p < 0.001) indicates that cortical perforation disrupts bone homeostasis. However, its effects vary by region, highlighting the need for region-specific imaging protocols to capture these nuances.

Unilocular lesions were associated with higher bone density in posterior (p = 0.024), superior (p = 0.005), and lingual (p < 0.001) regions than multilocular lesions. This finding supports the notion that unilocular lesions, such as radicular or dentigerous cysts, are generally less invasive and may preserve the surrounding bone better than multilocular lesions, such as ameloblastomas, which often exhibit aggressive growth patterns [3,18]. The higher bone density in these regions may reflect less destructive remodeling, making it a potential radiographic marker for less aggressive behavior.

Smooth-bordered lesions, often indicative of nonaggressive pathology, were associated with lower bone density in the anterior (p = 0.002), posterior (p < 0.001), inferior (p = 0.001), and lingual (p < 0.001) regions, as well as overall bone density (p < 0.001), but higher buccal density (p = 0.035). This suggests that smooth-bordered lesions, such as cysts, may cause diffuse bone loss due to slow expansive growth. In contrast, the buccal region may exhibit reactive thickening, possibly due to biomechanical adaptation [19]. This dual effect underscores the complexity of lesion border characteristics and their impact on bone density, emphasizing the need for a comprehensive radiographic evaluation.

The significant association between female sex and reduced inferior bone density (p = 0.001) may be attributed to hormonal influences, such as estrogen-related bone remodeling, or anatomical variations in the mandibular structure, as noted in previous literature [20]. This finding suggests that sex-specific factors should be considered in diagnostic and treatment planning processes, particularly for lesions affecting the inferior mandibular region.

The use of CBCT in this study was pivotal because its high-resolution, 3D imaging capabilities allowed precise quantification of bone density at lesion borders using HU. The strong correlation between CBCT VV and CT HU values, as reported by Cassetta et al. [9], validates CBCT as a reliable tool for assessing bone density. The standardized protocol, which included a voxel size of ≤0.2 mm and daily calibration with a CIRS Model 062M phantom, ensured measurement accuracy and reproducibility, as evidenced by high interobserver (ICC = 0.87) and intraobserver (ICC = 0.89-0.90) reliability. These methodological strengths enhance the reliability of the findings and support the use of CBCT as a standardized imaging modality for evaluating mandibular lesions [21].

Clinical implications

These findings have several clinical implications in the diagnosis and management of radiolucent mandibular lesions. First, the significant association between aggressive lesions and reduced bone density across most regions suggests that CBCT-based bone density measurements can serve as a quantitative technique for lesion aggressiveness. Clinicians can use these measurements to differentiate between benign and aggressive lesions, aiding in treatment planning. For instance, lesions with widespread bone loss and root resorption may require more aggressive surgical intervention, such as resection. In contrast, unilocular, smooth-bordered lesions with preserved bone density may be managed conservatively with enucleation or curettage.

Regional variations in bone density, particularly the paradoxical increase in buccal bone density in the absence of cortical perforation, highlight the importance of region-specific analysis in CBCT imaging. Clinicians should prioritize multiplanar reconstructions to comprehensively assess lesion borders, as buccal bone density may indicate compensatory remodeling, potentially influencing surgical approaches to preserve functional anatomy.

The sex-specific finding of reduced inferior bone density in women suggests that hormonal or anatomical factors may influence lesion behavior, necessitating tailored diagnostic and treatment strategies for female patients. For example, additional imaging or bone density assessments may be warranted in female patients with inferior mandibular lesions to monitor progression and guide intervention.

The high reliability of CBCT measurements in this study supports their integration into routine clinical practice for evaluating mandibular lesions. Dental radiologists and oral surgeons can leverage CBCT’s 3D visualization to assess lesion dimensions, margins, and spatial relationships with critical structures, such as the mandibular canal, thereby improving preoperative planning and reducing surgical complications.

Limitations

Despite its strengths, this study had several limitations. First, its retrospective design had the limitation of establishing causality between lesion characteristics and bone density changes. Prospective studies with longitudinal follow-up could elucidate the temporal relationship between lesion progression and bone density alterations. Second, the sample size of 125 patients, while adequate based on power calculations, might not fully capture the diversity of radiolucent mandibular lesions, particularly rare entities, such as odontogenic myxomas or malignant lesions. Larger, multicenter studies could enhance the generalizability of our findings.

Third, the study focused exclusively on radiolucent lesions, excluding mixed-density lesions, which may have limited the applicability of the findings to a broader range of mandibular pathologies. Future research should include mixed-density lesions to provide a more comprehensive understanding of variations in bone density. Fourth, while CBCT is highly effective for bone density assessment, its grayscale values may be influenced by scanner calibration and patient-specific factors such as bone thickness or soft tissue interference, potentially introducing measurement variability. Although calibration with a CIRS phantom mitigated this, standardized HU conversion protocols across CBCT systems are required.

Finally, the study did not account for systemic factors such as hormonal profiles or comorbidities that could influence bone density, particularly in female patients. Future studies should incorporate these variables to better understand their impact on lesion behavior and bone density.

## Conclusions

This study demonstrated that CBCT-based bone density measurements at the borders of radiolucent mandibular lesions provide valuable insights into lesion aggressiveness and regional bone interactions. Aggressive lesions, root resorption, and cortical perforation were strongly associated with reduced bone density, whereas unilocular and smooth-bordered lesions may preserve or even increase bone density in specific regions. These findings highlight the potential of CBCT as a diagnostic tool for differentiating benign from aggressive lesions with significant implications for treatment planning. Despite limitations, such as its retrospective design and focus on radiolucent lesions, this study underscores the importance of region-specific, quantitative imaging in maxillofacial radiology, paving the way for future research to refine diagnostic protocols and improve patient outcomes.
